# Trend of Daily Sports Participation in Korean Aged Population and Sports Policy: A Review of Research on Public Daily Sports Participation

**Published:** 2018-07

**Authors:** Misuk KIM, Sejeong PARK, Hyunjeong KIM, Eunsurk YI, Sangwan JEON

**Affiliations:** 1. Korea Institute of Sport Science, Korea Sports Promotion Foundation, Seoul, Korea; 2. Cultural Contents, Hankuk University of Foreign Studies, Seoul, Korea; 3. Dept. of Exercise Rehabilitation & Welfare, Gachon University, Incheon, Korea

**Keywords:** Korean aged population, Sports policy, Public daily sports participation

## Abstract

**Background:**

This study aimed to investigate the trend of physical activity and daily sports participation in the Korean aged population through the review of ‘Research on Public Daily Sports Participation’ published by the Ministry of Culture, Sports and Tourism. The main purpose was to suggest the best health and sports policy for the future.

**Methods:**

The result of the research conducted by the government was published 13 times in total from 1989 to 2015. The aged were defined as people in their 60s and 70s since 2006. Based on the research published 7 times from 2006 to 2015, this study analyzed the changes and the trend recognition of health status, physical activities, sports activity effects and environment in the aged population in South Korea.

**Results:**

Majority of the aged population was found to hardly recognize their health status, but positively aware of physical and sports activity effect, particularly that the sports facility environment has been improving. Therefore, it is encouraged to set up elderly-friendly routine sports environment to motivate their participation and consequently establish healthy exercise culture.

**Conclusion:**

This study has great significance as it suggests the direction of future health and sports policy by analyzing the trend of previous physical activities and daily sports participation among the aged population based on the government-published research.

## Introduction

Given that aging is a common phenomenon worldwide, multi-dimensional strategies are required particularly in South Korea that has become a super-aged society 10 years earlier than Japan. In 2015, the number of the aged, i.e. 65 years and above, in South Korea was 6540,000 (12.8%), and is expected to reach more than 10,000,000 and 18,180,000 by 2025 and 2045, respectively (1). Aging increases expenses, such as medical expenses and health insurance fee in particular, leading to great expectations in exercise, physical activity, physical education, and sports.

It was reported that regular physical activities are a strategy for health improvement and to increase empowerment and healthy life expectancy in senescence (2). Taking part in physical activities in the daily life in middlescence led to a healthier life in senescence (3). It was also reported that physical activities were safe and had positive effect on major cardiovascular and metabolic diseases, obesity, hurting from a fall, cognitive disorders, osteoporosis and muscular (4).

In South Korea, policies related to physical activities and exercises for the aged population has started to be visualized since 2000 when the country entered into the aged society (5). Public sports clubs, National Fitness Award, and program development and daily sports instructor projects are being supported by the Ministry of Culture, Sports and Tourism, which is authorized to control sports policy. Sports policies for the aged have been conducted as a part of daily sports policy vitalization since 1988 Seoul Olympic Games (6). The representative policies were as the follows: establishing Jangsu Sports College and gate ball stadiums, supporting social welfare facilities and daily sports activities in farming and fishing villages, and developing and distributing Health Practice for Homo-Hundred Projects under Roh Tae-woo government; holding Elderly Daily Sports Festivals, establishing gate ball stadiums in sports parks, and providing the elderly welfare facilities with sporting goods under Kim Young-sam government; establishing gate ball stadiums and providing the elderly social welfare with sporting goods under Kim Dae-jung government; establishing gate ball stadiums at si/gun/gu level, expanding Jangsu Sports College, enhancing daily sports outreach services and sporting goods support, and providing senior citizen’s centers and elderly welfare facilities with training service by sports instructor under Roh Moo-hyun government; and expanding outreach service for physical strength enhancement and daily sports class, distributing the General Harmonization Program, and expanding allocation of sports instructor to senior citizen’s centers under Park Geun-hye government (7–9).

However, as a result of reviewing the government policies to support the elderly mentioned above, it was difficult to find out middle- and long-term objective, programs reflecting rapidly changing needs of the elderly and consistency in projects except several cases. Although fundamental causes might vary by issue, there is a limit derived from that. There was no analysis of objective data on physical activities and sports participation in the elderly. In other words, it is because a comprehensive analysis on change was not properly conducted in and pattern of physical activities in the elderly by specific and systematic review of Research on Public Daily Sports Participation that is annually published by the Ministry of Culture, Sports and Tourism. As a critical basic data to scrutinize status of public daily sports participation in South Korea, the result of the research had been published a total of 13 times from 1989 to 2015, among which the result published since 2006 divided the elderly into 60s and 70s. The reason such critical data has not been properly utilized is probably the attention of the world of sports on the elderly which was not high or the importance of research as a national index was not recognized. Therefore, this study has great significance as the first study to suggest political direction for health and daily sports in the elderly, by analyzing the changes in and pattern of the citizens aged ≥60s’ in daily sports participation, based on the governmental annual research.

Systematic and supportive policies that are distinguishable from the policies implemented by the former governments need to be established to actively respond to rapidly increasing aged population. It is because the elderly’s participation in physical activities and daily sports was still low compared to the qualitative growth of available physical activities and daily sports. Compared to other developed countries, participation in regular exercise, club registration, experience in having training and exercise as a critical condition for maintaining physical strength in South Korea was found to be low. Therefore, the result of the research on recognition of health, recognition of physical and sports activity effect and satisfaction with physical and sports activity environment in the elderly is the critical key to establish policy of physical activity and sports in the elderly.

We aimed to investigate the trend of physical activity and daily sports participation in the Korean aged population through the review of Research on Public Daily Sports Participation published by the Ministry of Culture, Sports and Tourism.

## Materials and Methods

### Participants

This study was subjected to 9,000 Koreans at age ≥10 year. Among them, the elderly at 60s and ≥70s was selected by purposeful sampling.

### Analysis Method

This study used time series analysis on total 13 edits of Research on Public Daily Sports Participation from 1989 to 2015. However, for the aged population, data from 2006 to 2015 that classified people in their 60s and 70s since 2006, was used (10–16). Numerical calibration was conducted in non-investigated years (2007, 2009 and 2011) to examine long-term trend while calibration of moving average was used to show secular flow. In addition, data was arranged by obtaining common items, comprehensively combining similar items, sub-grouping short-term items, etc.

### Study Items

As policies of economy, culture, value and sports had changed from 2006 to 2015, a number of new study items, and newly added items were found of which sub-scale changed. Accordingly, common items were obtained in 2006, 2008, 2010, 2012, 2013, 2014, and 2015, a total of 7 times. In detail, these included recognition of the elderly’s own health status, conditions for maintaining health and physical strength, frequency of regular sports activities, immediate reason for start of sports activities, effect of sports activity participation on life, reason for inability to participate in sports activities, major type of sports activities, whether or not registration in sports club, type of sports club memberships, sports activity expenses, prerequisite for promotion of sports activity participation, and sports activity they want to try.

### Data Processing Method

This study used R (r-project.org) language for data reprocessing and numerical processing. R, as a statistical and visual program language, is the statistic method most frequently used in data analysis as an open source, which mutually finds out, controls and processes potential statistical and numerical fault.

## Results

### Recognition of Health Status

#### Degree of Health Status

Table 1 shows recognition of the aged population by their health status. It shows that 40% and more of the 60s had recognized their health status as ‘healthy’ since 2008 and such trend of recognition increased since 2010. Meanwhile, subjects who were negatively recognizing their health status as ‘not healthy’, ‘very weak’, etc. took approximately 10%.

**Table 1:** Recognition of health

***Yr***	***Very Healthy***		***Healthy***		***Moderately healthy***		***Not healthy***		***Very weak***	
	***60s***	***≥70s***	***60s***	***≥70s***	***60s***	***≥70s***	***60s***	***≥70s***	***60s***	***≥70s***
2006	4.3	4.3	26.2	26.2	30.3	30.3	32.1	32.1	2	7.2
2008	6.4	5.1	49.4	34.2	26.1	22	16.6	34.6	1.6	4.2
2010	3.9	1.8	43.3	25.2	31	31.1	20.3	35.6	1.5	6.3
2012	5.3	2.9	44.6	28.8	34	30.9	15	33	1.1	4.5
2013	2.9	2.2	46.6	29.3	38.8	38	10.9	28.1	0.7	2.5
2014	3.8	2	55.1	32.7	28.2	37.2	12.5	25.2	0.4	3
2015	4.3	1.5	53	24.5	30.9	37.6	11.2	32.5	0.6	4

Subjects who thought they were ‘not healthy’ among the ≥70s took more than 30% mean from 2006 to 2015. In 2013 and 2014, the percentage of subjects who responded to the question about health status as ‘not healthy’ decreased, while the percentage of subjects who responded as ‘moderately healthy’ increased.

#### Conditions for Maintaining Health and Physical Strength

Table 2 shows the conditions for maintaining health and physical strength among the aged population. ‘Regular sports activities’ was found to be the condition for health and physical strength ranked number 1 in people in their 60s from 2006 to 2015, which has been on downturn since 2010. ‘Regular meals and nutrition supplements’ has consistently taken an upturn, showing more remarkable in people in their ≥70s. In detail, ‘regular meals and nutrition supplements’ has been ranked number 1 in the ≥70s since 2016, taking an upturn with an average 40%, followed by ‘sufficient rest and sleep’ and ‘regular sports activities’ that was reported as the lowest rate.

**Table 2:** Conditions for maintaining health and physical strength

***Yr***	***Regular meals and nutrition***		***Regular sports activities***		***Sufficient rest and sleep***	
	***60s***	***≥70s***	***60s***	***≥70s***	***60s***	***≥70s***
2006	30	38.5	37.7	27.4	27.5	30.1
2008	31.45	39.2	35.45	30.6	29.75	26.75
2010	26.15	38.7	43.4	32.9	27.55	26.05
2012	33.5	42.45	33.5	29.25	28.6	25.9
2013	27.05	32.15	31.1	28.8	36.05	34.9
2014	34	41.9	33.15	25.35	28.7	29.05
2015	36.3	41.9	27.4	20.95	30.2	33.05

### Recognition of Physical Activity Effect on Health

#### Frequency of Regular Sports Activities

Table 3 shows frequency of regular sports activities in the aged population. According to the 8-year research since 2008, citizens in their 60s’ sports activity participation was found to be increasing. The rate of responders as ‘do not have sports activities at all’ was reported as 50% in 2008 and 2012, which has taken a rapid downturn since 2013.

**Table 3:** Frequency of regular sports activities

***Yr***	***Everyday***		***Once weekly***		***Twice weekly***		***3 times weekly***		***4 times weekly***		***5 times weekly***		***6 times weekly***		***2 to 3 times monthly***		***Not at all***	
	***60s***	***≥70s***	***60s***	***≥70s***	***60s***	***≥70s***	***60s***	***≥70s***	***60s***	***≥70s***	***60s***	***≥70s***	***60s***	***≥70s***	***60s***	***≥70s***	***60s***	***≥70s***
2008	12.7	13.5	6.5	4.3	5.4	2.8	9.7	5.9	4.8	1.5	4.3	3	2.7	1.8	3.3	2.4	50.5	64.8
2010	12.2	13.4	6.4	3.2	7.7	5.4	13.2	8.3	5.8	3.9	7.3	6.8	4.9	5	3.3	1	39.2	53
2012	8.4	10	7.5	4.3	5.5	3.9	11.7	8	3.3	4.5	7.4	5.7	3.5	5	3.5	1.4	49.3	57.2
2013	8.4	5.6	13.1	7.6	9.7	9.4	10.9	9.9	2.4	4.9	4.7	3.7	1.3	1.4	10.8	5.5	38.8	52
2014	10	10.7	9.9	5.6	8.9	6.9	11.3	8.4	5.5	3.9	5.4	4.2	6.9	5.2	13.1	10.7	28.8	44.3
2015	14	15.4	8.6	5.1	11.3	8.7	12.7	8.8	5.5	4.8	4.9	5.6	2.6	1.4	10.6	4.2	29.7	46.2

The rate of responders to ‘do not have sports activities at all’ was decreasing in the ≥70s. Even though a proportion of the aged who do not have sports activities at all among 70s was still 46.2% in 2015, which was still lower than average the 20% compared to the 60s, it was 18.6% less than that in 2008.

#### Immediate Reason for Start of Sports Activities

Table 4 shows immediate reasons for start of sports activities in the aged population

**Table 4:** Immediate reason for start of sports activities

***Yr***	***Good use of leisure time***		***Maintenance and improvement of health***		***Weight control***		***Stress relief***		***Self-satisfaction and self-realization***		***Personal relationship and social gathering***		***Personal pleasure***	
	***60s***	***≥70s***	***60s***	***≥70s***	***60s***	***≥70s***	***60s***	***≥70s***	***60s***	***≥70s***	***60s***	***≥70s***	***60s***	***≥70s***
2006	9.6	11.2	74.8	74.6	0.3	2.9	0.5	0	4.8	7.3	4	3.9	-	-
2008	8.8	9.7	71.1	76.4	9.2	3.4	4.6	3.8	0.9	0.4	1.8	1.7	2.8	3.8
2010	6.3	3.8	79.8	86.3	5.5	1.9	3.2	1	1	0.6	1.8	1.2	1.8	2.6
2012	5.7	4.2	72.5	77.8	10.4	7.1	4.4	1.3	1.9	1.7	1.6	1.8	2.6	4.8
2013	16.2	17.8	49	50	13.6	14.6	4.6	4.4	6	5.6	2.5	1.9	4.8	3.1
2014	19.5	15.3	59.8	62.5	5.6	6.4	3.4	1.4	2.5	3.8	3	1.7	3.2	3.2
2015	19.4	15.4	57.7	65.2	6.6	5.3	2.5	2.7	3.2	1.5	3.2	2	4.1	3

The largest proportion of people in their 60s was found to respond to the question of immediate reason for start of sports activities as ‘maintenance and improvement of health’. With an average responding rate of 66%, it shows their recognition of sports activity effect as ‘maintenance and improvement of health’.

#### Effect of Participation in Sports Activities on Life

Table 5 shows effect of the elderly’s participation in sports activities on their life. From 2006 to 2015, the cumulative rate of 75% responded to the question about effect of sports activity participation on life as positively as ‘moderate effect’ and ‘great effect’, with narrow increase-decrease width, among individuals in their 60s. The cumulative rate of 70% also responded as positively as ‘moderate effect’ and ‘great effect’, among the ≥70s.

**Table 5:** Effect of participation in sports activities on life

***Yr***	***No effect at all***		***Little effect***		***Some effect***		***Moderate effect***		***Great effect***	
	***60s***	***≥70s***	***60s***	***≥70s***	***60s***	***≥70s***	***60s***	***≥70s***	***60s***	***≥70s***
2006	0.1	0.2	2	2.9	19.4	26.2	70.6	64.8	7.8	6
2008	0	0.3	1.1	2.5	19.2	30	70.1	59.1	9.6	8
2010	0	0.2	0.5	0.8	18.7	28.4	70.2	62.6	10.6	8
2012	0	0	0.5	1	24.9	28.3	63.4	59.7	11.3	11
2013	0	0	0	0.1	9.9	14.9	74.7	70.8	15.4	14.2
2014	0.1	0	0.6	1.9	16.8	25.7	68.3	61.7	14.3	10.7
2015	0	0	0.5	0.9	20.1	27	69.2	62.9	10.2	9.2

#### Conditions and Status of Sports Activities Reason for Inability to Participate in Sports Activities

Table 6 shows reasons for inability to participate in sports activities in the aged population. Among responses obtained from the 60s, ‘lack of time’ was ranked number 1, followed by ‘aging and lack of physical strength’ ranked number 2, with average responding rate of 20%, which was considered as the main reason.

**Table 6:** Reason for inability to participate in sports activities

***Yr***	***Lack of affordability***		***Lack of information***		***Lack of time***		***Aging and lack of physical strength***		***Lack of interest***		***No exercise partner***		***No place and facility***		***Lack of program***		***Income level***	
	***60s***	***≥70s***	***60s***	***≥70s***	***60s***	***≥70s***	***60s***	***≥70s***	***60s***	***≥70s***	***60s***	***≥70s***	***60s***	***≥70s***	***60s***	***≥70s***	***60s***	***≥70s***
2006	2.2	0.5	0.9	0	39.2	41.6	14.5	1.9	-	-	0.6	0.5	2.2	7.9	-	-	-	-
2008	2.6	1.5	2.1	1.3	37.1	22.9	25.1	48.4	-	-	3.8	1.5	4	4.2	-	-	-	-
2010	2.2	1.4	1.2	1.1	37	18	28.2	57.5	-	-	3.8	3.3	4.7	2.7	-	-	-	-
2012	5.5	3.8	2.5	1.6	34.4	14.6	16.1	41.5	29.7	27.8	4.8	4.3	4.6	4.5	1.6	1.6	-	-
2013	6.6	4.5	2.5	2.4	26.3	10.8	25.2	56	25.1	16.5	3.8	1.9	5.8	3.7	2.2	1.3	2.5	2.4
2014	5.9	2.8	2.4	3.6	41.4	18.4	14.8	38.2	7.7	15.1	3	1.4	5.8	5.2	2.1	0.4	8.3	6.5
2015	5.6	3.3	1.2	1.8	33.9	14.2	19.7	43.2	11.4	12.2	1.3	1.9	3	4.2	2.3	1.1	7.8	6.2

Meanwhile, ‘aging and lack of physical strength’ was predominant in the ≥70s while the responding rate of ‘lack of time’ that used to approximately 40% in 2006 largely decreased and maintained as approximately 15%.

#### Major Type of Sports Activities

Table 7 shows major types of sports activities usually practiced by the aged population. The major types of exercise usually practiced by the 60s was walking at approximately 50% and hiking at average 20%. Walking and hiking neither involve expenses nor require specific technique and training. As same to 60s, walking was ranked number 1 in ≥70 at approximately 66%, while hiking was ranked number 2. People at the age of ≥70 were found to prefer walking, free exercises, yoga, etc. to active sports such as hiking, weight training and cycling.

**Table 7:** Major type of sports activities

***Yr***	***Hiking***		***Swimming***		***Weight training***		***Cycling***		***Yoga***		***Walking***		***Free exercises***	
	***60s***	***≥70s***	***60s***	***≥70s***	***60s***	***≥70s***	***60s***	***≥70s***	***60s***	***≥70s***	***60s***	***≥70s***	***60s***	***≥70s***
2006	24.9	18.5	4	1	2.6	1	1.9	2.4	1.4	1	-	-	-	-
2008	21.7	8.9	4.4	5.1	7.2	4.2	3.2	6.3	1.8	2.5	52.2	62	4.6	4.6
2010	23.3	8.9	4	1.3	6.3	4.2	5.5	4.5	0	0	56.1	69.6	3	8.3
2012	18	8.8	5	2.3	4.2	1.8	6.1	2.5	1.4	2.8	53	65.5	1.9	5
2013	26.8	19.2	6	4.7	3.7	4.4	1.5	1	1.7	2.9	51.4	64.8	4.4	9.2
2014	15.6	7.4	4.4	3.8	4.8	1.4	3.7	3.5	3.5	3.3	49.1	65.8	-	-
2015	19.6	8.7	4.4	3.8	2.8	0.7	4	1.6	2.5	3.4	49.9	68.2	3.5	5

#### Sports Activity Expenses

Table 8 shows sports activity expenses in the aged population. The number 1 ranked response was found to be ‘no expenses at all’ in individuals in their 60s; in particular, 60% among them did not spend any money at all from 2010 to 2012, which gradually declined since then. The rate of responders of “no sports activity expenses at all” was found as 62% in ≥70s, which tended to slightly decrease.

**Table 8:** Sports activity expenses

***Yr***	***No expenses at all***		***Less than KRW 30,000***		***Less than KRW 30,000 - 60,000***		***Less than KRW 60,000 - 100,000***		***Less than KRW 100,000 - 150,000***		***KRW 150,000 or more***	
	***60s***	***≥70s***	***60s***	***≥70s***	***60s***	***≥70s***	***60s***	***≥70s***	***60s***	***≥70s***	***60s***	***≥70s***
2006	54.3	70.7	12.3	10.7	15.4	11.2	4.7	1.5				
2008	59	68.6	14.4	13.1	17.1	12.7	3.7	0.8	5.1	3.4	0.7	1.3
2010	50	61.7	28.8	29.7	14	6.7	2.4	0.3	3	1	1.8	0.6
2012	60.1	72.6	11.7	16.9	14.4	6.5	6.1	2.4	3.5	0.9	4.2	0.7
2013	39.1	5.7	14.2	14.3	22.9	21.9	7.7	5.1	9.3	5.9	6.9	1.9
2014	40.6	56.7	15.8	14.2	20.8	20.7	8.4	1.2	10.7	4.2	3.7	2.9
2015	45.6	57.6	17.1	21.7	21.7	14.7	4.7	1.4	7.3	3.3	3.6	1.5

#### Prerequisite for Promotion of Sports Activity Participation

Table 9 shows prerequisite for promotion of sports activity participation in the aged population. ‘Increased time for sports activities’ was selected by more than 30% of individuals in their 60s. In addition, the rate of responders to ‘increased interest in sports activities’ was in an upward trend as more than 10%. Other than such external (environmental) motivations, items such as the internal motivations of responders as ‘improved health status’ as well as ‘increased income level’ increased by more than 15% and nearly 10%, respectively. ‘Improved health status’ was preferentially chosen by those aged 70and above, exceeding 30%, while ‘increased time for sports activities’ was found to be an external (environmental) motivation similar to those citizens in their 60s.

**Table 9:** Prerequisite for promotion of sports activity participation

***Yr***	***Increased time for sports activities***		***Increased interest in sports***		***Improved health status***		***Expanded access to sports facilities***		***Increased income level***		***Expanded sports program***		***Sports activity expense affordability***		***Presence of exercise partner***		***Expanded access to sports activity information***	
	***60s***	***≥70s***	***60s***	***≥70s***	***60s***	***≥70s***	***60s***	***≥70s***	***60s***	***≥70s***	***60s***	***≥70s***	***60s***	***≥70s***	***60s***	***≥70s***	***60s***	***≥70s***
2013	33.6	27.2	8.2	7.6	18.1	30.3	18.1	12.6	7.4	5.7	0.3	1.2	7.7	8.3	3.6	3	1.9	3.4
2014	37.4	15.6	9.7	11.5	12.7	30.7	13.7	12.7	9.8	8.3	3	3	7.3	9.5	3.1	4.9	2.6	3.2
2015	30.2	21	14.1	9.5	17.5	34	7.9	7.7	9.6	9.6	4.6	4	6.8	7.7	4.7	2.9	3.9	2.5

#### Preferred Sports Activity

Table 10 and 11 show sports activities preferred by the aged population. 15% of the 60s choose ‘hiking’ and ‘swimming’ as their favorite sport activities. 8% were found to favor yoga, gate ball and golf, among which gate ball was in a downward trend. Meanwhile, ‘gate ball’, ‘hiking’ and ‘swimming’ were found to be preferred by exceeding 10% of ≥70s, which were in an upward trend. ‘Yoga’ was preferred by an average 7.2% while preference for ‘fishing’ and ‘golf’ was slightly increasing.

**Table 10:** Preferred sports activity (60s)

***Yr***	***Swimming***	***Hiking***	***Yoga***	***Gateball***	***Golf***	***Dance sport***	***Fishing***	***Cycling***	***Weight training***	***Aerobics***	***Badminton***	***Tennis***
2006	13.4	9.7	7.9		12.9				1.1	4.2	2.4	2.3
2008	15.9	12.7	9	6.3	3.2	3.1		2.2	3.9	2.7	2.3	1.5
2010	16.5	17.8	6.8	5.2	4.1	3.1	2	3.3	3.1	1.6	1.5	1.4
2012	13.5	17.9	7.7	10.7	2.1	4.8		2.8	2.6	1.8	3.6	0.8
2013	11.8	11.6	6.7	7.2	6.1	4.4	4.7	4.5	3.2	4.5	1.3	1.5
2014	15.3	14.5	7.7	7	6.8	5.4	5.7	2.4	3.7	3.3	2.7	0.9
2015	15.7	13.5	7.7	5.8	3.5	6.6	4.9	3.3	3	2.3	2.5	1

**Table 11:** Preferred sports activity (≥70s)

	***Gateball***	***Hiking***	***Swimming***	***Yoga***	***Fishing***	***Aerobics***	***Golf***	***Dance sport***	***Cycling***	***Weight training***	***Badminton***	***Tennis***
2006	-	9.6	8.7	2.6	-	9.7	9.6	-	-	3.8	0.2	4.8
2008	9.8	9.5	11.3	9.5	-	1.8	2.7	2.2	1.5	0.7	1.2	0.4
2010	13.8	10.7	12	6.6	1	0.8	0.6	2	2	-	0.5	0.4
2012	15.6	13.6	9.9	5.5	-	1.2	1	2	1.1	0.5	2	0.1
2013	9.7	12.8	6.7	10.6	5.2	5.7	3.4	2.8	5.9	1.7	2.7	0
2014	19.8	13.8	14.9	7.4	5.1	2.3	2.9	3.1	1.5	1.1	2.2	0.1
2015	16.2	13.5	15.3	7.9	5.6	1.7	1.6	4.5	1.7	1.8	1.6	0.5

## Discussion

This study is aimed to investigate the trend of physical activity and daily sports participation in the Korean aged population through the review of Research on Public Daily Sports Participation published by the Ministry of Culture, Sports and Tourism. Therefore, these discussions are based on the result of physical activity and daily sports participation in the aged groups, the 60s and ≥70s for 9 years reported in research.

Totally 40% of the 60s were found to recognize their health status as ‘healthy’ since 2008. The rate of responders of ‘moderate’ in 60s was maintained at 30% range, implying that they have a potential to positively recognize their health status by controlling their condition with no disease diagnosed. Meanwhile, there was no big difference in the rate of responders as ‘not healthy’ and ‘moderate’ in the individuals aged 70 and above between 2006 and 2015, indicating no big difference in the degree of self-recognition of health in the 70s in general. Therefore, they need to raise self-recognition of health in the 70s via intensive management. The “Rate of regular sports activities” in relation to ‘conditions for maintaining health and physical strength’ under the health status recognition item tended to decrease in both the 60s and 70s while “regular meals and nutrition supplements” tended to increase, particularly in the ≥70s to average 40% since 2006. It might be because of its effectiveness in a short time, in addition to the factors such as the influence of media such as a number of home shopping channels, as well the information sharing through word-of-mouth in the elderly at the centers for senior citizens and community relief centers.

The second discussion was about ‘frequency of regular sports activities’ under the “recognition of physical activity effect for health” item. There was an increase in physical activity participation in the 60s for 8 years since 2008, as the rate of responders of “no sports activities at all” has rapidly decreased since 2013. Meanwhile, in 2015, the rate of responders of “no sports activities at all” was 46.2% in the 70s, which was 18.6% less than that in 2008. The population that had sports activities at least once a week had increased, in addition to approximately 5% increase in proportion of the ≥70s who have exercise every day since 2013. 60% of both the 60s and 70s found the sports value as “maintenance and improvement of health”, followed by good use of leisure time, weight control, self-satisfaction and self-reliance, and personal pleasure. For effect of sports activity participation on life, more than 70% of both age groups provided positive responses as ‘moderate effect’ and ‘great effect’. It is especially significant in terms of providing the 70s who responded as ‘not highly recognizing my health condition’ with sports activities.

The third discussion was about “reason for inability to participate in sports activities” under the “conditions and status of sports activities” item. It was found that the 60s and 70s made different choice in this regard. 60s predominantly responded as “lack of time’, followed by ‘aging and lack of physical strength’. In the 70s, “aging and lack of physical strength” took the largest proportion. It implies that the reason for inability to participate in sports activities could be found from the internal cause of an individual. Meanwhile, decreased complaints about problems on circumstances of sports activities such as lack of facilities, infrastructure, programs and information indicate that external circumstances have been gradually improved. In both aged groups, “walking” and “hiking” were found to be “major types of sports activities”. Those activities are characterized as low cost, no specific technique, and high accessibility. There was no big difference in major types of sports activities between the 60s and 70s. Participation in weight training was low in the 70s, suggesting that active intervention is required. It is because the exercise programs for the aged group need to prevent potential injuries caused by physical imbalance due to decreased muscular strength. For “sports activity expenses”, 60% of 60s responded as “no expenses at all” from 2010 to 2012 while 20% was found to spend less than KRW 30,000–60,000 or less than KRW 30,000. Meanwhile 62% of the 70s responded as “no sports activity expenses at all” while approximately 20% responded as “less than KRW 30,000”, suggesting that both the 60s and 70s were participating in low cost sports activities. For “prerequisite for promotion of sports activity participation”, the 60s responded with “increased time for sports activities” and “increased interest in sports activities” while the 70s responded as “improved health status”, suggesting that measures to enhance internal motivation are required for both groups. For “preferred sports activity”, the 60s responded with “hiking and swimming” while the 70s responded as “gate ball, hiking, and swimming”. Interest in “golf” were found to be in an upward trend in the minority of both groups, and preference for strenuous sports was confirm to decrease in the 70s rather than in the 60s.

Analysis of results of each research might be available for meaningful comparison with overseas cases. In Japan, with emergence of the term lifelong sports since 1990, sports programs customized for the aged had been run while Basic Sports Plan was established in 2012, aiming at the number of daily sports non-participants (who had never participated in sports activities for last 1 year) as “0” (zero). Elderly sports and bodily exercises promotion projects, new sports projects (Shuffle board, Indiaca, Petanque, Bound Tennis, Disk Golf, Check Ball, Soft Bolley Ball, and Ground Gold), elderly health classes (muscular strength balance, cognitive disease prevention, daily habitant disease prevention and dementia prevention, etc.), prevention by caring projects are in running.

In Germany, there are various programs comprehensively run by German Olympic Sports Confederation. “Healthiness from Age 50 (Richtig Fit ab 50)”, “Exercise Network 50 Plus”, “Exercise Program 70 Plus”, “Active and Fit”, projects for prevention of injuries from fall, projects for elderly exercise training, etc. are representative. In addition, programs and projects for elderly are running by state-level sports councils; for example, “Active to 100” by German Gymnastics Association, “Exercise Beginner Support” by Hesse Sports Council, “Generation Integration Project - Sports with the Young and Elderly” by Westphalia Sports Council, etc. As mentioned earlier, although self-recognition of physical activities, exercises and sports activities in the old age is high in South Korea, consistent supporting policies by central and local governments are still needed (17–19). Accordingly, the best way to increase physical activity participation in the aged population might be securing safety of activities by running interesting and divergent programs.

## Conclusion

This study suggests the best policy for health and sports policy as the following, as a result of examine health status, effect of sports activities, and understanding of environment in the aged population as discussed earlier: 1) ‘Enhancement of participating motivation’ in physical activities and sports; 2) ‘Development of sports participation environment’ for easy access by the aged population; and 3) ‘Expansion of sports value’.

## Ethical considerations

Ethical issues (Including plagiarism, informed consent, misconduct, data fabrication and/or falsification, double publication and/or submission, redundancy, etc.) have been completely observed by the authors.
